# Minireview on the Relations between Gut Microflora and Parkinson's Disease: Further Biochemical (Oxidative Stress), Inflammatory, and Neurological Particularities

**DOI:** 10.1155/2020/4518023

**Published:** 2020-02-05

**Authors:** Ovidiu-Dumitru Ilie, Alin Ciobica, Jack McKenna, Bogdan Doroftei, Ioannis Mavroudis

**Affiliations:** ^1^Department of Research, Faculty of Biology, “Alexandru Ioan Cuza” University, Carol I Avenue, no. 11, 700505 Iasi, Romania; ^2^Leeds Teaching Hospitals NHS Trust, Great George St., Leeds LS1 3EX, UK; ^3^Faculty of Medicine, University of Medicine and Pharmacy “Grigore T. Popa”, University Street, no. 16, 700115 Iasi, Romania; ^4^Origyn Fertility Center, Palace Street, no. 3C, 700032 Iasi, Romania; ^5^Laboratory of Neuropathology and Electron Microscopy, School of Medicine, Aristotle University of Thessaloniki, 541 24 Thessaloniki, Greece

## Abstract

The aetiology of Parkinson's disease (PD) is a highly debated topic. Despite the progressive increase in the number of patients diagnosed with PD over the last couple of decades, the causes remain largely unknown. This report is aimed at highlighting the main features of the microbial communities which have been termed “the second brain” that may be a major participant in the etiopathophysiology of PD. It is possible that dysbiosis could be caused by an overactivity of proinflammatory cytokines which act on the gastrointestinal tract as well as infections. The majority of patients who are diagnosed with PD display gastrointestinal symptoms as one of the earliest features. In addition, an unbalanced cycle of oxidative stress caused by dysbacteriosis may have the effect of gradually promoting PD's specific phenotype. Thus, it seems that bacteria possess the ability to manipulate the brain by initiating specific responses, defining their capability to configure the human body, with oxidative stress playing a pivotal role in preventing infections but also in activating related signalling pathways.

## 1. Introducing Some Basic Aspects about (Gut) Microflora: The Unseen Companion—Functions and Future Perspectives

The Human Genome Project (HGP) identified that the human DNA consists of 3 billion base pairs, respectively, 20,500 genes and nearly double the number of coding proteins, and 1.4 million single-nucleotide polymorphisms (SNPs) when it was officially completed in 2003 [1]. The emergence of the Human Microbiome Project (HMP) in 2008 stimulated a significant increase in further research in commensal bacteria, culminating in an increase in the number of studies regarding the relationships between intestinal flora and the etiopathophysiology of neurodegenerative and psychiatric disorders [2].

It has been well established that all microorganisms that populate our body are grouped into four major ecosystems. The greatest number of associations is being gathered at the level of the digestive tract, with a density of 10^14^. This is approximately ten times more entities than the total number of cells involved in the structure of an individual. The human microbiome possesses over one hundred and fifty times more bacterial genes and a biomass production weighing equivalent to that of the human brain. The average total number of microbes populating a reference male with a normal constitution is close to forty trillion. Increased numbers of pluricellular organisms could be viewed as ideal amphitrions, alongside our tenants (large collections of archaea, bacteria, fungi, and viruses), ensuring an invisible endo- and exoskeleton thanks to this symbiotic bond [3–6].

The human body harbours between five hundred and one thousand species which are subsequently divided into three enterotypes: *Ruminococcus*, *Bacteroides*, and *Prevotella*. Next-generation sequencing protocols are widely used to both identify and to characterise these communities [7–9]. The gastrointestinal tract (GI) hosts trillions of microbes, with each community exerting beneficial or harmful effects upon the normal development of the central nervous system (CNS). However, dysbiosis is associated with an increased susceptibility to various diseases. The aforementioned echoes the “repair your gut and you will repair your brain” [10–12].

Without a shadow of doubt, it is clear that we have evolved in tandem with this microworld throughout the millennia, with the microflora becoming an integrated part of any human being. Joshua Lederberg coined the term “microbiome” in order to describe the collection of commensal, symbiotic, and pathogenic entities. Antonie van Leeuwenhoek is the first person who analysed the major differences at the faecal and oral level in the 1680s [13]. They saw the light of day about four billion years ago, long before the appearance of the first man and oxygenation of the earth [14, 15].

Gastrointestinal (GI) microbiota fulfil crucial functions with the aim of maintaining metabolic homeostasis such as direct inhibition of pathogen overgrowth, development of enteric protection, biosynthesis of vitamins, energy modulation, and immunological and xenobiotic effects. In addition, they aid drug metabolism by producing essential small bioactive molecules like short-chain fatty acids (SCFAs) (butyrate, acetate, and propionate), bile acids, choline, amino acids and phenolic derivatives (AAA), polysaccharide A (PSA), indole, and nicotinic, aminoethylsulfonic, or retinoic acids, precursors involved in mediating interactions with the human body by keeping the integrity of neurohormonal axes [16–19].

**Figure 1:**
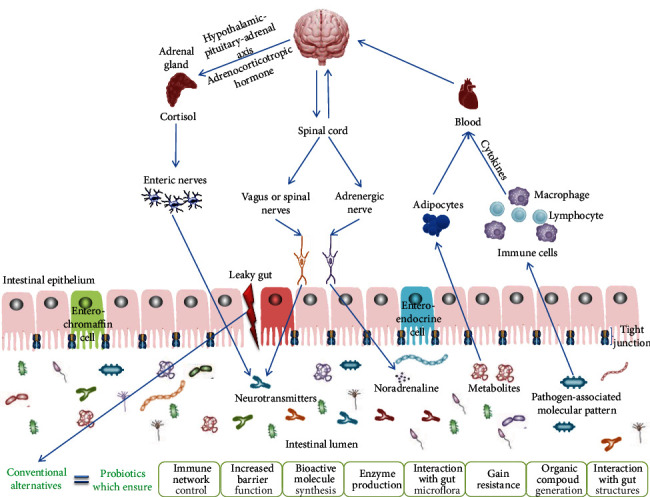
Principal pathways in the gut-brain circuit. Deregulation of microbial associations will induce deficient signals by initiating specific responses and subsequently inhibiting their whole functionality. Probiotics are usually used to reestablish these discrepancies (adapted from [20]).

Unfortunately, the relationship between GI flora and the brain is insufficiently understood. The influence that the gut flora exerts on the local organs in the immediate vicinity, as well as on those located distally, is taking place through a variety of routes, for example, immune, enteric, and neural pathways. Thus, the gut-brain axis (GBA) could be defined as a dense network formed by cells from the enteric, peripheral, and central nervous system in association with the hypothalamic-pituitary-adrenal (HPA) axis (Figure 1) [20–22].

Historically, there has been a tendency to believe that each one of us possesses the same gut microflora, a theory that has been proven to be only partially true. There are interindividual and intergenerational variations in the microbiome which is influenced by the changing environment, nutritional factors, and genetic contributions. The literature suggests numerous clues that sustain this hypothesis; for example, not even twins harbour the same composition of microflora [23]. The similarities are even less prevalent amongst siblings, but nonetheless, in very small percentages, there are similarities in flora composition even in unrelated individuals [24, 25].

One of the most important factors in shaping the normal neonatal enteric colonization of microflora is the delivery method. Although the gut of an unborn baby is theoretically sterile in the mother's womb, the development of the neonatal microbiota is initiated by the neonate transversing the birth channel, where there is a subsequent exposure to a large amount of maternal microbial communities which shapes the microbiota of the infant. By analysing different cohorts via computational research, a set of specific bacterial genes were classified in limited habitats (e.g., placenta), evidence that is sustained after the analysis of meconium samples where it was revealed that colonization may be initiated *in utero* [26–29].

On the other hand, a recent publication contradicts these findings. The study design was aimed at determining whether preeclampsia, small for gestational age (SGA), and spontaneous preterm birth (PTB) were correlated with the existence of bacterial signatures in the placenta. Authors concluded that the placenta is devoid of such populations but nevertheless provides favourable conditions for pathogenic species such as *Streptococcus agalactiae*; this species is prevalent in almost 5% of the total samples collected before the beginning of procedures [30].

There are a large amount of species facultatively anaerobic (enterobacteria and enterococci) that are found in the GI of children, with their whole existence depending on the dietary supply, thus creating propitious conditions for the evolution of anaerobic microbes. However, the childhood microbiota may also be influenced by other environmental factors, such as exposure to healthcare facilities and other children culminating in complex and dynamic microbiota [31, 32].

In this context, natural birth is supported in order to maintain the balance between beneficial and harmful microorganisms. However, in the last few decades, the number of caesarean sections (C-section) has increased dramatically which is worrying. Women are not adequately informed about the risk to the baby through a C-section delivery method, possibly predisposing the infant to a series of epidemiological illnesses. Obesity, allergies, anaphylactic reactions to asthma, and autoimmune diseases are a few examples of conditions which may be influenced by the commensal bacteria and thus the delivery method. It is evident that C-section indirectly promotes various diseases through the effect of the neonatal microbiota [33, 34].

The delivery mode creates a disbalance amongst gram-positive and gram-negative species, which can be beneficial to certain species such as *Lactobacillus*, *Bifidobacterium*, *Eubacterium*, and *Bacteroides*, to the detriment of those pathogens like *Clostridium*, *Campylobacter*, *Staphylococcus*, *Shigella*, *Shiga toxin-producing Escherichia coli*, *Acinetobacter*, and *Escherichia coli* [35–38]. Breastfeeding has the potential to reestablish this balance, alongside conventional alternatives, for example, syn-, pre-, and probiotics, which have proven to be powerful tools with extraordinary potential in restoring metabolic homeostasis [39].

Continuing on with this concept, there are numerous other factors which have been shown to promote the development of antibiotic resistance in certain cases. This is conducted by activating the human resistome which has only been recently discovered. Some examples of potential factors that can promote antibiotic resistance include the maternal diet, overall neonatal health, age, environmental factors, and prolonged exposure to antibiotics [40–42].

In a recent study, researchers discovered that the resistome (gigantic tank of Antibiotic Resistance Determinants (ARDs)) of infants born prematurely is already preformed because of the antibiotics used in order to prevent different infections, with gene-drug-type studies becoming possible through Mobile-CRISPRi. By using dedicated techniques, they revealed distinctive patterns and an emerging multidrug resistance of *Enterobacteriaceae* during and after hospitalization [43, 44]. Ciprofloxacin, an antibiotic usually administered to treat bacterial infections, has a long-term effect upon bacterial diversity even after half a year since after the end of the treatment [45].

## 2. The Relevance of Gut Microflora in Parkinson's Disease Pathogenesis/Pathophysiology

Parkinson's disease (PD) is the second most common neurodegenerative, progressive, and debilitating disorder of the parkinsonism spectrum, clinically manifesting through symptoms of bradykinesia, stiffness, trembling, and postural instability. It is characterised by a perpetual loss of dopaminergic neurons from the substantia nigra pars compacta (SNpc) and of cholinergic neurons from the posterior motor nucleus of the vagus, along with a continuous accumulation and aggregation of *α*-synuclein in the central nervous system (CNS) [46].

There is no typical age of onset of this disorder; hence, it was thought to be the result of exposure to various exogenous factors, but genetics has proven to also play an important role in its pathogenesis. Distinct genes and loci have been identified; however, the aetiology of this disorder remains largely uncertain [47, 48].

The incidence has increased exponentially since the nineties. In 2016, more than six million people were diagnosed with PD, and it became the second most common neurodegenerative disorder worldwide [49].

A relationship amongst enteric neurons and gut microflora has been reported due to new discoveries around toll-like receptors, proteins with a key role in the innate immune system [50, 51], and their modulation potential upon the HPA axis [52], followed by a further production of chemicals involved in the brain's optimal functioning [53]. There is new evidence concerning the importance of toll-like receptor 4 in mediating neuroinflammatory states, resulting in the disruption of intestinal flora, while rotenone KO-treated mice had a reduction of specific symptomatology [54]. In rotenone models, chronic stress induces a deregulation of HPA which may culminate in dysbacteriosis, characterised by a significant reduction in the number of species belonging to the genus *Bifidobacterium*, to the detriment of *Escherichia coli*. Prolonged exposure leads to an increased intestinal permeability which creates a “leaky gut,” dysosmia, and colitis by inducing specific neuroanatomical and neurochemical changes [55–59].

**Figure 2:**
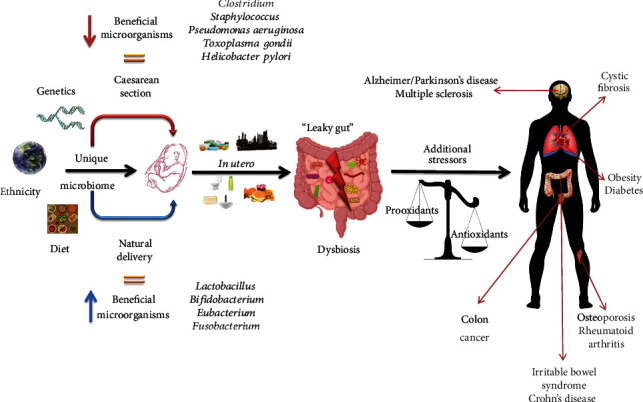
Schematic representation of the cycle in which the delivery mode and daily habits could gradually create dysbiosis and cause chronic conditions.

An imbalance in the host's microbiota (dysbacteriosis) can manifest in the development of low-grade inflammation, cellular degeneration, and an imbalance of cellular energy followed by an increasing oxidative stress (OS) state [60]. An overactivity of clusters of differentiation 4, 1, and 17 [61] will inhibit the responses of peripheral immune cells [62] which will disturb the integrity of the blood-brain barrier (BBB) and its role against bacterial lipopolysaccharides (LPS) and other toxins [63, 64]. Dysbiosis can cause numerous disorders, and one of these conditions is PD (Figure 2) [65].

The importance of the influence of microflora on the BBB is demonstrated by the species *Enterococcus faecalis* and *Eggerthella lenta*, both of which have the ability to metabolise levodopa, which is the principal drug that is administered to people who have PD. It was shown that L-dopa did not cross the BBB in order to release dopamine, resulting in a much shorter route because of these microorganisms [66]. According to the literature, microbial tyrosine decarboxylase (TDC) is a bacterial amino acid which has the ability to restrict the release of dopamine thus inhibiting the effects of levodopa [67].

According to the Food and Agriculture Organization of the United Nations, which is in agreement with the guidelines established by the World Health Organization, probiotics can be defined as “living microorganisms in adequate doses ensuring a shield to the host by improving the general state of health.” Unlike probiotics, prebiotics are food supplements recommended for stimulation of the growth and/or activity of those that are beneficial [68]. For example, *in vitro* observations led to the conclusion that *Bacillus* sp. JPJ can produce levodopa from 4-hydroxyphenylalanine which is subsequently converted to dopamine [69].

In addition, mixtures of lactic bacteria obtained from fermented products restore the integrity of the microbiota by enhancing the gut barrier following an exposure to antibiotics in certain intervals [70]. There are novel techniques which facilitate the manipulation of the gut microflora by suppressing pathogens in the epithelium and intestines, in order to regulate the activity of immune cells [71]. Finally, synbiotics are a mixture of the two categories mentioned earlier, with the main aim of increasing the duration of life and settlement of those already existing in the GI [72].

Faecal Microbiota Transplantation (FMT) is a treatment that facilitates the reconstruction of the gut flora whereby faecal matter from a healthy donor is donated to a patient thereby changing the underlying microflora. This treatment is used in the treatment of resistant *Clostridium difficile* infections. Microbial Transfer Therapy (MTT) is a similar protocol to FMT, both of them demonstrating their potentials in treating metabolic deficiencies [73, 74].

The differences in the clinical manifestation of PD mean that the management needs to be individualised. For example, chronic idiopathic constipation (CIC) is encountered in PD subjects and can be associated with anorectal and colonic dysmotility [75]. FMT intervention caused motor impairment in mice and humans, promoting a reduction in *Lachnospiraceae* and *Ruminococcaceae* strains [76]. In progeroid mice, however, FTM reduced both morbidity and mortality. These observations can also be applied in human patients, where a reduction in *Proteobacteria* in parallel with increased *Verrucomicrobia* concentrations was documented [77].

The discovery of bacteriophages with more recent clustered regularly interspaced short palindromic repeats (CRISPR) and the associated nuclease 9 has led to an array of possibilities to manipulate the human microbiome [78, 79]. This technique started from the discovery of foreign sequences of DNA from viruses that were incorporated into bacteria. Those sequences confer immunity against future interactions with viruses and have shown extraordinary potential in manipulating the human genome. This technique is used to influence the genome of those resistant to antibiotics [80] or metabolise various drugs with the aim of integrating this system into conventional products.

Surveys published over the years support the concept of the gut-brain network and *vice versa*; some of them regarding a better understanding of influence exerted by the microbiome on PD patients are summarised in Table 1.

## 3. Gut Infections as a Promoter in Parkinson's Disease

The implications of *Helicobacter pylori* in dyspepsia and gastritis are well documented. However, in PD, it appears to be associated with an increased severity of motor functions [91], by inhibiting and controlling dopamine levels in the brain [92]. Antimicrobial treatments against *H. pylori* improved absorption of levodopa [93]. However, no clear conclusions can be drawn regarding the implications of *H. pylori* in PD because there has been a lack of clinical trials. It is certain that the presence of *H. pylori* in the GI tract could interfere in different PD treatment regimens by initiating autoimmune or inflammatory reactions [94–96].

There are increased populations of intestinal bacteria in patients with PD with estimates alluding to an overpopulation of greater than 50% when compared to the intestinal microbiota populations of patients without PD [97, 98]. There are frequent treatment failures of patients treated for PD, with a recent randomised trial suggesting that possible eradication of surplus will not affect the pharmacokinetics of L-dopa [99]. Another study analysed the role of infection as a cause for PD by analysing the serum antibody titre through ELISA. They analysed the antibody titres to common pathogens including cytomegalovirus, herpes simplex virus type 1, *Helicobacter pylori*, Epstein-Barr virus, *Borrelia burgdorferi*, and *Chlamydophila pneumoniae*. They conclude that the bacterial and viral burden was independently associated with PD [100].

Matheoud et al. [101] provided a pathophysiological model following an infection with *Citrobacter rodentium*. PINK1 is a repressor of the immune system and as a result is engaged in mitochondrial antigen presentation and autoimmune mechanisms that elicit the establishment of cytotoxic T cells in the brain. Any alteration of PINK1 can induce tumorigenesis, while parkin, encoded by the PARK2 gene, is usually involved in early-onset parkinsonism.

In a 16S rRNA NGS and quantitative polymerase chain reaction analysis, *Influenza A* virus induced insignificant changes after the infection at the level of the tracheobronchial tree with a minor reactivity of the immune system, but in the intestines, there was a depletion of the bacterial composition with an increase in the host defence peptides (HDPs) in Paneth cells and a tear of the mucous membrane [102].

In 2005, Nerius et al. [103] initiated the largest prospective German study (*n* = 228,485 individuals with an average age of 50 or older), with the main objective of determining the prevalence of PD in patients who have previously suffered from common gastrointestinal infections (GIIs). The study identified that 77.9% did not suffer from any GI infections, while 22.1% reported previous infections. The results suggested that the predisposition to PD is significantly higher (*p* < 0.001) in people who have suffered from GIIs when compared to the control group.

The gradual dysfunction of the enteric nervous system (ENS) amplifies the probability of small intestinal bacterial overgrowth (SIBO). There are extensive cross-sectional studies which highlight an increased prevalence of SIBO in PD patients compared with the control groups [104]. This is supported by a study in which the authors revealed that SIBO is a condition that could be treated with an appropriate treatment regime, such as rifaximin 200 mg 3 times per day for 1 week, which improves not only gastrointestinal symptoms but also motor fluctuations [105].

In addition, the clinical features of irritable bowel syndrome (IBS), which include bloating and flatulence, are also common symptoms in PD patients, while constipation or rectal tenesmus does not define the clinical panel of IBS but is however present in patients with PD [104]. However, a recent study revealed an unusual case in which early PD was treated by using antibiotics and colchicine. Moreover, these drugs improved constipation and diminished the PD-like symptoms [105].

## 4. Gastrointestinal Deficiencies? The Links between the Gut Communities' Structure and Parkinson's Disease

Aside from motor dysfunctionalities, patients with PD also manifest metabolic disturbances with half of them suffering from constipation prior to the onset of other clinical features. This suggests a possible link between early gastrointestinal problems and later evolving stage of PD [106]. Over the last half decade, a limited number of studies were conducted, with the aim of exploring the impact of the gastrointestinal microbiota in the prodromal and early stages of PD [107, 108]. Since gastrointestinal deficiencies like constipation significantly contribute toward the morbidity in PD, a recent clinical study has identified that regular intakes of *Lactobacillus casei Shirota* could diminish such disturbances and bowel movement in PD [109]. Vitamin D3 prevented deterioration in the Hoehn and Yahr stage in PD patients, and vitamin D exerted beneficial activity both *in vivo* and *in vitro* against 6-hydroxydopamine [110, 111]. Another study identified that following a twenty-four-week administration of riboflavin, there was a significant increase in the motor capacity in PD patients by normalising vitamin B6 status and after all red meat was eliminated. Symptomatology did not reappear even if the treatment was interrupted for several days; this suggests that low levels of vitamin B6 may promote motor impairment [112].

Variations in the patient's inclusion/exclusion, statistical, and molecular criteria and bioinformatic methodologies amongst the studies are presented in Table 1. The majority of the studies focused on the bacterial 16S ribosomal DNA amplicon sequence, in particular next-generation sequencing (NGS) protocols at the species, genus, and phylum level, 1 quantitative polymerase chain reaction using preselected taxa and 1 metagenomic shotgun sequencing. The cohorts had varied sizes, with the smallest group containing a total of 24 individuals and the largest one having 197 individuals. In each one, particularities were noted, both in PD and in healthy controls, and together, the overall characteristics in faecal gastrointestinal flora composition were distinct. It is not certain whether or not these changes are the cause or result of GI dysfunctionality.

Heintz-Buschart et al. [82] propose that the gastrointestinal microbiome (GM) alteration most likely precedes the development of motor symptoms in PD. The genus *Ralstonia* was responsible for proinflammatory reactions in the mucosa compared to the controls [76]. In some cases, it was observed that there was an alteration of several metabolic pathways (lipopolysaccharide and ubiquinone and bacterial emission and xenobiotic metabolism or tryptophan) [76, 83, 90]. In addition, low levels of faecal SCFAs were reported in PD patients by a theoretically deteriorating enteric nervous system [88].

Barichella et al. [84] evaluate atypical parkinsonism, more specifically, the composition of the gut in multiple system atrophy (MSA) and Steele-Richardson-Olszewski syndrome, in which some bacterial taxa have undergone changes similar to PD, while drug-naïve persons displayed low abundance of *Lachnospiraceae*, almost 43% reduction identified in contrast to *Bifidobacterium* [81].

The gram-negative *Prevotella* population was diminished to almost 78% compared to controls with 38.9% specificity in PD [85]. Dysbacteriosis that occurred in Chinese patients promoted features such as disease duration, levodopa equivalent doses (LED), and cognitive impairment, while in the German cohort, alpha and beta analysis highlighted a similar pattern with the exception of the *Barnesiella* genus and *Enterococcaceae* family who were present in abundance [86, 87]. Furthermore, cellulose-degrading bacterial concentration is lower, whereas putative pathobionts are dramatically increased [89].

## 5. The Relationship between Oxidative Stress and Gut Microbiota in the Context of PD

One of the most defining capabilities of nicotinamide adenine dinucleotide (NAD) as a ubiquitous metabolite is its involvement in the production of energy. It therefore follows that mitochondrial dysfunction was associated with various disorders, including PD. Cumulative learnings highlight the NAD role in processes like neuroprotection as well as playing a role in maintaining the integrity of DNA by activating specific mechanisms against oxidative stress. NAD also contributes toward the synthesis of adenosine triphosphate (ATP), calcium signalling, gene expression, and apoptosis. Three NAD-consuming enzymes, poly (ADP-ribose) polymerase (PARPs), sirtuins (SIRT), and CD38/157, secure the integrity of DNA [113, 114]. *In vivo* imaging data indicates that aging is the main factor involved in the build-up of insults, implicitly resulting in diseases such as diabetes and cardiovascular, metabolic, and neurological problems, or may result from a depletion or restriction of vitamin B3 [115].

In contrast, NAD usually participates in the processes that contribute toward energy homeostasis, generally associated with the subsequent production of reactive oxygen species (ROS). Its phosphorylated derivative NADP, a result of NAD kinase (NADK), plays a vital role in maintaining antioxidant defences, but in some tissues, it can serve as a cofactor in the reactions that generate free radicals [116–118]. There are pros and cons to the importance of oxidative stress in processes like apoptosis of dopaminergic neurons and in the accumulation of insults in PD [119, 120].

Wistar male rats were used in Y-maze and shuttle box tasks. This is a procedure that is used to determine the neurotoxic effect of 6-hydroxydopamine (6-OHDA) in experimental rodents in which the ventral tegmental area (VTA) or SN is targeted using a defined apparatus and protocol. Modifications were observed in both procedures in VTA and SN, with 6-OHDA affecting their cognitive sphere for a short duration of time, in parallel with a depletion of SOD and GPx; this approach further supports a link amid OS and PD [121]. Moreover, it was examined whether OS in the hippocampus has any implications upon memory by injecting two different unilateral doses of LPS (memory impairment action) into the SN of adult male Wistar rats. Rodents were examined in a pergolide-induced rotational behaviour test to determine the amount of damage inflicted upon nigrostriatal dopaminergic neurons. In the hippocampus of LPS-treated rats, levels of malondialdehyde were significantly higher compared with those in controls which were measured in Y-maze (within was noted correlations between behavioural deficiencies as indexes for OS) and radial arm maze tasks [122]. In a quite similar manner, all of the aforementioned compounds and tests were combined into one study, with behavioural deficiencies being more pronounced only in LPS- and LPS+6-OHDA-treated rats [123].

Even though our cells are equipped with mechanisms which counteract the accumulation of insults, the fact that we are strictly aerobic organisms can have severe repercussions on the state of health and the reduction of molecular oxygen to O_2_ and H_2_O during the cellular respiration process that leads to the synthesis of adenosine triphosphate (ATP). This promotes the production of free radicals, with 20% of the total oxygen supply consumed by the brain being converted into ROS. These reactive species generated by nicotinamide adenine dinucleotide phosphate oxidase (NOX) and nitric oxide synthase (NOS) perform functions like resistance against infections and the activation of various signalling pathways [124, 125].

NADPH is in excess compared to NADP whose ratio is much lower than 1; in this context, apart from the NAD/NADH ratio, cells maintain two opposite redox pairs with NADP/NADPH in a continuous reductive state, and this redox stability is compatible with the NADPH role in biosynthesis and detoxification with oxygen. NADPH is a key reducing substrate for transforming oxidised glutathione into reduced glutathione as a protective element against toxicity of ROS. An increased ratio of NADH/NAD is associated with a petulant production of reactive oxygen species and the inhibition of *α*-ketoglutarate dehydrogenase due to a mitochondrial dysfunction and the inability of antioxidant enzymes such as superoxide dismutase (SOD) and glutathione peroxidase (GPx) to maintain balance [126, 127].

Besides ROS, cumulative surveys highlight the importance of reactive nitrogen species (RNS), entities generated as a result of interactions between superoxide (O_2_^−^) and nitric oxide (NO), resulting in large amounts of peroxynitrite. NO, produced by NOS, commonly exists under three isoforms, known as endothelial NOS (eNOS), neuronal NOS (nNOS), and inducible NOS (iNOS), which can be found in glial cells [128–131]. Peroxynitrite has the ability to induce DNA fragmentation and lipid peroxidation because of its oxidative structure and even dose-dependent impairment independently of dopamine normal cycle and death [129, 130]. *In situ* hybridisation and immunohistochemistry of postmortem brain tissue revealed high expression of iNOS and nNOS in PD patients which further highlights the role of NO. In the substantia nigra, the gliosis is linked to an upregulation of the iNOS, while it is linked to the inhibition of nNOS against cytotoxicity of MPTP (neurotoxin). Neuronal death still remains an enigma, but with the current evidence, it can be concluded that oxidative stress and mitochondrial dysfunctions are interconnected, especially at the level of respiratory chain, highlighted by a petulant production of reactive oxygen species which leads finally to apoptosis in PD [132, 133].

Dopamine (DA) (excitatory and inhibitory role of synaptic transmission) as a construct produced from DA neurons can in turn be a source of OS due to its unstable nature selectivity for SNpc (*substantia nigra pars compacta*) which undergoes self-oxidation in order to form dopamine quinones and free radicals, reactions catalysed by oxygen, enzymes, or metals [134, 135]. Interestingly, with an excessive amount of cytosolic DA outside of the synaptic vesicles, this neurotransmitter is easily metabolised by monoamine oxidase (MAO), a participant in the regulation of DA levels by monoamine oxidase A (MAO-A), localised in catecholaminergic neurons [136].

Alternatively, in degeneration that occurs in PD or aging, monoamine oxidase B (MAO-B) becomes the predominant enzyme that metabolises DA and can be found in glial cells and then taken up by astrocytes [137]. In transgenic mice, the wilful induction of this enzyme in astrocytes had as result a selective and progressive loss of nigral dopaminergic neurons [138]. It has been shown that DA quinones have the ability to shape proteins which subsequently may be involved in PD pathophysiology, for example, *α*-synuclein, parkin, protein deglycase DJ-1, and ubiquitin carboxy-terminal hydrolase L1, in *α*-synuclein DA quinone modifying its monomer by promoting the conversion to a cytotoxic protofibril form [139]. These quinones can be oxidised into aminochrome, whose redox cycle capacity ultimately causes the depletion of NADPH and the generation of superoxide. This is subsequently transformed into neuromelanin (brain pigment that might play a role in neurodegeneration), occurring within the SNpc [140, 141]. Taking into account the circuit of PD and that the dorsal motor nucleus of the vagus nerve (DMnX) is the primary hive cluster to *α*-synuclein, *in vivo* models provide additional clues regarding the participation of oxidative stress into the spreading of a “mutated” *α*-synuclein within and outside the CNS by promoting cell and protein interrelations. They show that cholinergic neurons are very sensitive to the accumulation of reactive oxygen species (ROS) [142]. Accumulation of *α*-synuclein, encoded by the SNCA gene, is also a risk factor for PD, containing inclusions in the enteric nervous system and posterior motor nucleus of the vagus [143, 144], determining overinflammatory reactions, intestinal permeability, and oxidative stress [145, 146].

Following the analysis of 117 tissue samples and 161 from controls, biopsies revealed an accumulation of *α*-synuclein at the level of the various oesophageal tunics and ganglia, with implications of this protein usually involved in neurotransmitter release being much more complex [147, 148]. Also, the 465-residue E3 ubiquitin ligase parkin is covalently modified by dopamine becoming insoluble, leading to ubiquitin E2 ligase inactivation, in the SN, with catechol-“mutated” parkin being observed in patients with PD, but not in other regions of the brain [149].

The modification of ubiquitin carboxy-terminal hydrolase L1 (UCH-L1) and protein deglycase DJ-1 by dopamine quinones was observed in dopaminergic cells, but also in mitochondria, and because of the cysteine residue they possess, quinones are responsible for the inactivation of these enzymes [150]. In a transgenic murine model ((Thy-1)-h[A30P]-*α*-synuclein) with SOD2 haplodeficiency, at 1 year and 4 months, exhibiting significant features of synucleinopathy compared full SOD2 control, the results indicate that an elevated level of OS could mediate the progression of PD [142]. Kim et al. [151] tested Braak's theory that *α*-syn could spread into the brain from the gut via the vagus nerve. They injected preformed *α*-syn fibrils in a novel gut-to-brain mouse model and found that *α*-syn is dispersed first into the posterior motor nucleus of the vagus and then in caudal portions of the rhombencephalon. Furthermore, specific symptoms were present temporarily, but truncal vagotomy and deficiency of *α*-syn prevented its further spreading.

In the model gut bacterium *Enterococcus durans* (MTCC 3031), oxidative stress induced by C_6_H_4_(CO)_2_C_2_H(CH_3_) and H_2_O_2_ deregulates the redox ratio (55% for menadione and 28% for H_2_O_2_) by decreasing folate synthesis of these gram-positive bacteria, known to play an important role against colorectal cancer [152]. By measuring the amount of hydrogen production for both gram-negative and gram-positive bacteria, it is speculated that the bacteria participate in the progression of PD [153].

Pathogen-associated molecular patterns (PAMPs) are conserved motifs that activate pattern recognition receptors (PRRs) found on the surface of diverse pathogens and induce ROS. Lipopolysaccharides (LPS) produced by bacteria are usually recognised by these PRRs which generate downstream signals and activate the NF-*κ*B pathway and induce inflammatory responses [154]. In the case of commensal bacteria, the lipopolysaccharides produce and release formyl peptides which are recognised by formyl peptide receptors (FPRs), a class that belongs to the G protein-coupled receptors; many signalling cascades utilise these receptors for converting a large variety of external stimuli (agonist neurotransmitters, ions, and hormones) into intracellular responses, perceiving and stimulating ROS production [155]. Due to the fact that our intestines house distinct cell types by initiating specific responses, ROS produced by mucosa-resident cells or by recruiting innate immune cells are crucial for an optimal antimicrobial activity. An unbalanced ROS synthesis through activating certain gene variants and upregulation of oxidases or of a mitochondrial dysfunction is associated with Crohn's disease or ulcerative colitis. In this way, the abnormal profiles of intestinal flora may lead to inflammation of the intestines often seen in people with inflammatory bowel disease (IBD) [156, 157].

## 6. Conclusions

It can be concluded that there are numerous factors (antibiotics, diet, birth mode, or stress) which gradually promote the onset of enteric dysbacteriosis which may trigger disorders of the CNS. There are relatively few studies that highlight the relationship between intestinal flora and PD; researchers argue that these limitations will be overcome due to the fact that the human microbiome is currently the main barrier to the emergence of personalised medicine. Oxidative stress is an integrative component to the function of all organisms, regardless of the current status (homeostasis or disease). This paper summarised most of the existing evidence in the literature, and it can be concluded that the wider implications of the human microbiome are complex and requires further research to improve the current understanding of the mechanisms underlying neurodegenerative disorders like Parkinson's disease.

## Figures and Tables

**Table 1:** 

Number of patients	Type of study	Differences at the family (left) and genus (right) level in PD cases		Differences at the family (left) and genus (right) level in healthy control cases		Reference
75 PD 45 HC	V3 16S rRNA gene sequencing Illumina HiSeq	Bifidobacteriaceae Eubacteriaceae Aerococcaceae Desulfovibrionaceae	—	Streptococcaceae Methylobacteriaceae Comamonadaceae Halomonadaceae Hyphomonadaceae Brucellaceae Xanthomonadaceae Lachnospiraceae Actinomycetaceae Sphingomonadaceae Pasteurellaceae Micrococcaceae Brevibacteriaceae Gemellaceae Idiomarinaceae Intrasporangiaceae Methanobacteriaceae	—	[81]

76 PD 78 HC	V4 16S rRNA gene sequencing and whole metagenome sequencing Illumina HiSeq	Verrucomicrobiaceae	Akkermansia Clostridium XIVb Anaerotruncus	—	—	[82]

197 PD 130 HC	16S rRNA gene sequencing Illumina MiSeq	Bifidobacteriaceae Lactobacillaceae Christensenellaceae Verrucomicrobiaceae	Bifidobacterium Lactobacillus Akkermansia	Lachnospiraceae Pasteurellaceae	Blautia Roseburia Faecalibacterium	[83]

193 PD (39) drug naïve 22 PSP and MSA 113 HC	V3-V4 16S rRNA gene sequencing Illumina MiSeq	Verrucomicrobiaceae Enterobacteriaceae Christensenellaceae Lactobacillaceae Coriobacteriaceae Bifidobacteriaceae	Akkermansia Parabacteroides Ruminococcus Oscillospira	Lachnospiraceae	Roseburia	[84]

72 PD 72 HC	V1-V3 16S rRNA gene sequencing Pyrosequencing	Lactobacillaceae Verrucomicrobiaceae Bradyrhizobiaceae Clostridiales	—	Prevotellaceae	—	[85]

45 PD 45 HC	V3-V4 16S rRNA gene sequencing Illumina MiSeq	—	Clostridium IV Aquabacterium Holdemania Sphingomonas Clostridium XVIII Butyricicoccus Anaerotruncus	—	—	[86]

29 PD 29 HC	V1-V2 16S rRNA gene sequencing Illumina MiSeq	Lactobacillaceae Barnesiellaceae Enterococcaceae	—	—	—	[87]

34 PD 34 HC	qPCR	Enterobacteriaceae	Akkermansia muciniphila Bifidobacterium	Prevotellaceae	Faecalibacterium prausnitzii Lactobacillaceae Enterococcaceae	[88]

38 PD 34 HC	V4 16S rRNA gene sequencing Illumina MiSeq	Bacteroidaceae Clostridiaceae Verrucomicrobiaceae	Bacteroides Oscillospira Akkermansia	Lachnospiraceae Coprobacillaceae	Blautia Coprococcus Dorea Roseburia	[76]

24 PD 14 HC	V3-V5 16S rRNA gene sequencing Illumina MiSeq	Enterobacteriaceae Veillonellaceae Erysipelotrichaceae Coriobacteriaceae Streptococcaceae Moraxellaceae Enterococcaceae	Acidaminococcus Enterococcus Streptococcus Acinetobacter Escherichia-Shigella Megamonas Megasphaera Proteus	—	Blautia Faecalibacterium Ruminococcus	[89]

31 PD 28 HC	Metagenomic shotgun sequencing Illumina HiSeq	—	Akkermansia Unknown bacteria and Firmicutes	—	Prevotella Eubacterium	[90]

PD = Parkinson's disease; HC = healthy control; MSA = multiple system atrophy; PSP = progressive supranuclear palsy.
